# Crystal structure of *catena*-poly[[tetra­aqua­magnesium]-μ-(di­hydrogen hypodiphosphato)-κ^2^ *O*:*O*′]

**DOI:** 10.1107/S2056989015012037

**Published:** 2015-06-27

**Authors:** Mimoza Gjikaj, Madeline Haase

**Affiliations:** a Institute of Inorganic and Analytical Chemistry, Clausthal University of Technology, Paul-Ernst-Strasse 4, D-38678 Clausthal-Zellerfeld, Germany

**Keywords:** crystal structure, hydrogen bonding, chain structure, hypodiphosphate, magnesium

## Abstract

The crystal structure of the title compound, [Mg(H_2_P_2_O_6_)(H_2_O)_4_]_*n*_, is built up from (H_2_P_2_O_6_)^2−^ anions bridging Mg^2+^ cations into chains extending parallel to [011]. The Mg^2+^ ion is located on an inversion centre and is octa­hedrally coordinated by the O atoms of two (H_2_P_2_O_6_)^2−^ anions and four water mol­ecules. The centrosymmetric (H_2_P_2_O_6_)^2−^ anion has a staggered conformation whereby the tetra­valent phospho­rus atom is surrounded tetra­hedrally by three O atoms and by one symmetry-related P atom. A three-dimensional O—H⋯O hydrogen-bonded network of medium strength involving the P—OH group of the anion and the water mol­ecules is present.

## Abstract 2

[Mg(H_2_P_2_O_6_)(H_2_O)_4_] is the first alkaline earth hypodiphosphate to be structurally determined. It consists of (H_2_P_2_O_6_)^2−^ anions that are bridged by Mg^2+^ cations into a chain structure. Water mol­ecules complete the octa­hedral coordination sphere of the metal and built up a three-dimensional hydrogen-bonding network.

## Chemical context   

A considerable number of alkaline metal hypodiphosphates have been characterized in the last few years (Szafranowska *et al.*, 2012 ▸; Wu *et al.*, 2012 ▸; Gjikaj *et al.*, 2012 ▸, 2014 ▸). Until today, the described alkaline metal hypodiphosphates have only been of academic inter­est, with the exception of ammonium and sodium di­hydrogenhypodiphosphates (Collin & Willis, 1971 ▸). The acidic solutions of sodium di­hydrogen­hypo­diphosphate are used for the gravimetric immobilization of uranium(IV) as U_2_P_2_O_6_·3H_2_O and UP_2_O_7_ (Bloss *et al.*, 1967 ▸). Furthermore, ammonium di­hydrogenhypodiphosphate finds a use as a flame retardant (Ruflin *et al.*, 2007 ▸), and its ferroelectricity has recently been discovered (Szklarz *et al.*, 2011 ▸).

The alkaline earth metal hypodiphosphates were first described by Salzer (1878 ▸). Ca_2_P_2_O_6_·2H_2_O and BaH_2_P_2_O_6_·2H_2_O were first synthesized by Palmer (1961 ▸), but structural data of hypodiphosphates of the alkaline earth metals are still missing. Here, we report the synthesis and the crystal structure of [Mg(H_2_P_2_O_6_)(H_2_O)_4_].

## Structural commentary   

**Figure 1:**
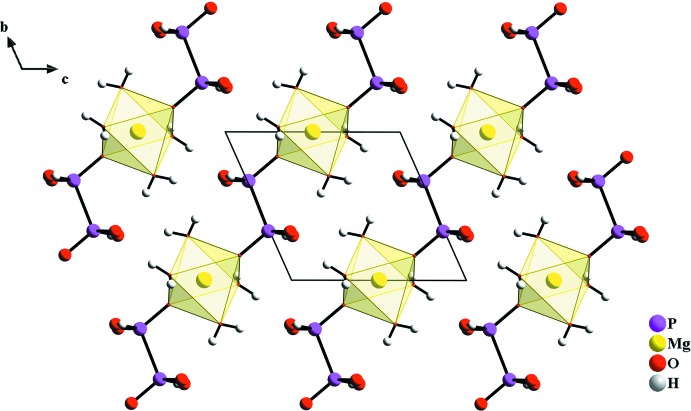
The crystal structure of the title compound, viewed along [100], showing the chain architecture.

**Figure 2:**
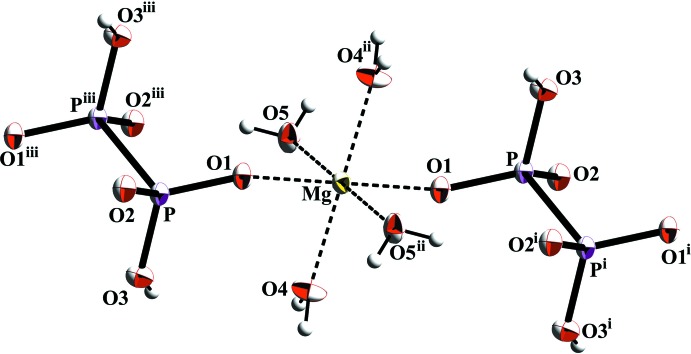
The mol­ecular entities in the title compound with atom labels and displacement ellipsoids drawn at the 50% probability level. [Symmetry codes: (i) −*x*, −*y* + 1, −*z*; (ii) −*x*, −*y* + 2, −*z* + 1; (iii) *x*, *y* + 1, *z* + 1.]

The principal building units in the crystal structure of [Mg(H_2_P_2_O_6_)(H_2_O)_4_] are [MgO_6_] octa­hedra and (H_2_P_2_O_6_)^2−^ anions, forming chains extending parallel to [011] (Fig. 1 ▸). In the chains, each Mg^2+^ cation is bridged by two anions (Fig. 2 ▸). The Mg^2+^ ion is located on an inversion centre and is octa­hedrally coordinated by two (H_2_P_2_O_6_)^2−^ anions and by four water mol­ecules with Mg—O bond lengths ranging from 2.0580 (17) to 2.0646 (18) Å. In the (H_2_P_2_O_6_)^2−^ anion, which is located about an inversion centre and has a staggered conformation, the tetra­valent P atom is surrounded by three O atoms and one symmetry-related P atom with a P—P distance of 2.1843 (12) Å and P—O distances ranging from 1.5013 (16) to 1.5855 (16) Å. All bond lengths and angles of the hypodiphosphate anion are well within the expected ranges (Szafranowska *et al.*, 2012 ▸; Gjikaj *et al.*, 2014 ▸) and are comparable to those found for *M*_2_P_2_O_6_·12H_2_O (*M* = Co and Ni; Hagen & Jansen, 1995 ▸; Haag *et al.*, 2005 ▸).

## Supra­molecular features   

**Figure 3:**
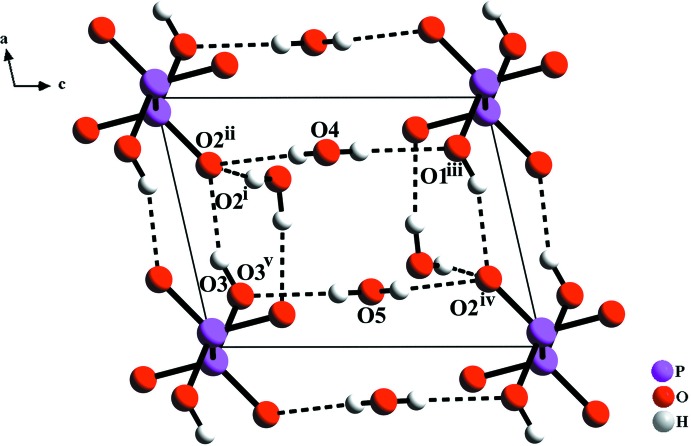
The hydrogen bonds between (H_2_P_2_O_6_)^2–^ anions and water mol­ecules in the title compound. The symmetry codes are as in Table 1 ▸.

The crystal structure of [Mg(H_2_P_2_O_6_)(H_2_O)_4_] exhibits a three-dimensional hydrogen-bonded network, in which the (H_2_P_2_O_6_)^2–^ anions are joined into ribbons along [100] by centrosymmetric pairs of PO3—H3⋯O2 hydrogen bonds (Table 1 ▸ and Fig. 3 ▸). The O⋯O distances between the (H_2_P_2_O_6_)^2–^ anions and water mol­ecules located between the ribbons range from 2.786 (3) to 2.829 (3) Å), indicating hydrogen bonds of medium strength (Table 1 ▸). These values agree very well with those reported for Rb_2_H_2_P_2_O_6_·2H_2_O (Wu *et al.*, 2012 ▸).

## Synthesis and crystallization   

Disodium di­hydrogenhypodiphosphate was prepared according to Leininger & Chulski (1953 ▸). An aqueous solution of hypodi­phospho­ric acid was obtained by passing a saturated solution of disodium di­hydrogenhypodiphosphate through a cation-exchange resin (Dowex 50WX2 50–100). About 40 ml of an aqueous solution of hypodi­phospho­ric acid (H_4_P_2_O_6_) were collected in the pH range 1.5–3.5 and subsequently added to magnesium carbonate (117 mg) at room temperature. Colourless block-shaped crystals of the title compound were obtained after several days at 278 K.

## Refinement   

Crystal data, data collection and structure refinement details are summarized in Table 2 ▸. All hydrogen atoms were located in a difference Fourier map and were refined isotropically without restraints.

## Supplementary Material

Crystal structure: contains datablock(s) I, New_Global_Publ_Block. DOI: 10.1107/S2056989015012037/wm5175sup1.cif

Structure factors: contains datablock(s) I. DOI: 10.1107/S2056989015012037/wm5175Isup2.hkl

CCDC reference: 1408335

Additional supporting information:  crystallographic information; 3D view; checkCIF report

## Figures and Tables

**Table 1:** Hydrogen-bond geometry (, )

*D*H*A*	*D*H	H*A*	*D* *A*	*D*H*A*
O3H3O2^i^	0.79(4)	1.94(4)	2.687(2)	157(3)
O4H4*A*O2^ii^	0.82(3)	2.00(3)	2.817(2)	169(3)
O4H4*B*O1^iii^	0.85(4)	1.94(4)	2.786(2)	173(3)
O5H5*A*O2^iv^	0.75(4)	2.03(4)	2.768(3)	165(3)
O5H5*B*O3^v^	0.77(4)	2.08(4)	2.829(3)	165(4)

Symmetry codes: (i) 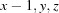; (ii) 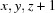; (iii) 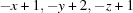; (iv) 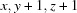; (v) 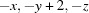.

**Table 2:** Experimental details

Crystal data	
Chemical formula	[Mg(H_2_P_2_O_6_)(H_2_O)_4_]
*M*_r_	256.33
Crystal system, space group	Triclinic, *P* 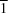
Temperature (K)	223
*a*, *b*, *c* ()	5.1486(15), 6.595(2), 7.096(2)
, , ()	112.31(2), 98.55(2), 98.28(2)
*V* (^3^)	215.09(11)
*Z*	1
Radiation type	Mo *K*
(mm^1^)	0.61
Crystal size (mm)	0.28 0.25 0.23

Data collection	
Diffractometer	Stoe IPDS-II
Absorption correction	Numerical (*X-SHAPE* and *X-RED*; Stoe Cie, 1999 ▸, 2001 ▸)
*T*_min_, *T*_max_	0.843, 0.869
No. of measured, independent and observed [*I* > 2(*I*)] reflections	2193, 799, 739
*R*_int_	0.057
(sin /)_max_ (^1^)	0.609

Refinement	
*R*[*F* ^2^ > 2(*F* ^2^)], *wR*(*F* ^2^), *S*	0.036, 0.094, 1.15
No. of reflections	799
No. of parameters	81
H-atom treatment	All H-atom parameters refined
_max_, _min_ (e ^3^)	0.60, 0.53

Computer programs: *X-AREA* (Stoe Cie, 2002 ▸), *SHELXS97* and *SHELXL97* (Sheldrick, 2008 ▸), *DIAMOND* (Brandenburg, 2012 ▸), *PLATON* (Spek, 2009 ▸) and *publCIF* (Westrip, 2010 ▸).

## supplementary crystallographic information

### Crystal data

**Table:**

[Mg(H_2_P_2_O_6_)(H_2_O)_4_]	*Z* = 1
*M**_r_* = 256.33	*F*(000) = 132
Triclinic, *P*1	*D*_x_ = 1.979 Mg m^−^^3^
Hall symbol: -P 1	Mo *K*α radiation, λ = 0.71073 Å
*a* = 5.1486 (15) Å	Cell parameters from 3841 reflections
*b* = 6.595 (2) Å	θ = 3.2–25.7°
*c* = 7.096 (2) Å	µ = 0.61 mm^−^^1^
α = 112.31 (2)°	*T* = 223 K
β = 98.55 (2)°	Block-shaped, colourless
γ = 98.28 (2)°	0.28 × 0.25 × 0.23 mm
*V* = 215.09 (11) Å^3^	

### Data collection

**Table:**

Stoe IPDS-II diffractometer	799 independent reflections
Radiation source: fine-focus sealed tube	739 reflections with *I* > 2σ(*I*)
Graphite monochromator	*R*_int_ = 0.057
ω–scans	θ_max_ = 25.7°, θ_min_ = 3.2°
Absorption correction: numerical (*X-SHAPE* and *X-RED*; Stoe & Cie, 1999, 2001)	*h* = −6→6
*T*_min_ = 0.843, *T*_max_ = 0.869	*k* = −8→8
2193 measured reflections	*l* = −8→8

### Refinement

**Table:**

Refinement on *F*^2^	Primary atom site location: structure-invariant direct methods
Least-squares matrix: full	Secondary atom site location: difference Fourier map
*R*[*F*^2^ > 2σ(*F*^2^)] = 0.036	Hydrogen site location: inferred from neighbouring sites
*wR*(*F*^2^) = 0.094	All H-atom parameters refined
*S* = 1.15	*w* = 1/[σ^2^(*F*_o_^2^) + (0.0594*P*)^2^ + 0.0577*P*] where *P* = (*F*_o_^2^ + 2*F*_c_^2^)/3
799 reflections	(Δ/σ)_max_ < 0.001
81 parameters	Δρ_max_ = 0.60 e Å^−^^3^
0 restraints	Δρ_min_ = −0.53 e Å^−^^3^

### Special details

**Table:**

Geometry. All e.s.d.'s (except the e.s.d. in the dihedral angle between two l.s. planes) are estimated using the full covariance matrix. The cell e.s.d.'s are taken into account individually in the estimation of e.s.d.'s in distances, angles and torsion angles; correlations between e.s.d.'s in cell parameters are only used when they are defined by crystal symmetry. An approximate (isotropic) treatment of cell e.s.d.'s is used for estimating e.s.d.'s involving l.s. planes.
Refinement. Refinement of *F*^2^ against ALL reflections. The weighted *R*-factor *wR* and goodness of fit *S* are based on *F*^2^, conventional *R*-factors *R* are based on *F*, with *F* set to zero for negative *F*^2^. The threshold expression of *F*^2^ > σ(*F*^2^) is used only for calculating *R*-factors(gt) *etc*. and is not relevant to the choice of reflections for refinement. *R*-factors based on *F*^2^ are statistically about twice as large as those based on *F*, and *R*- factors based on ALL data will be even larger.

### Fractional atomic coordinates and isotropic or equivalent isotropic displacement parameters (Å^2^)

**Table:**

	*x*	*y*	*z*	*U*_iso_*/*U*_eq_	
P	0.05636 (10)	0.66957 (9)	0.00780 (8)	0.0119 (2)	
Mg	0.0000	1.0000	0.5000	0.0118 (3)	
O1	0.1302 (3)	0.8332 (3)	0.2329 (2)	0.0158 (4)	
O2	0.2710 (3)	0.6732 (3)	−0.1158 (2)	0.0167 (4)	
O3	−0.2044 (3)	0.6995 (3)	−0.1200 (2)	0.0180 (4)	
O4	0.3263 (3)	0.9689 (3)	0.6850 (3)	0.0233 (4)	
O5	0.2204 (3)	1.2973 (3)	0.5191 (3)	0.0207 (4)	
H3	−0.345 (8)	0.697 (6)	−0.086 (6)	0.041 (9)*	
H4A	0.326 (6)	0.895 (5)	0.756 (5)	0.019 (7)*	
H4B	0.491 (7)	1.027 (5)	0.699 (5)	0.024 (7)*	
H5A	0.229 (6)	1.410 (6)	0.605 (6)	0.026 (8)*	
H5B	0.220 (7)	1.323 (6)	0.422 (6)	0.041 (10)*	

### Atomic displacement parameters (Å^2^)

**Table:**

	*U*^11^	*U*^22^	*U*^33^	*U*^12^	*U*^13^	*U*^23^
P	0.0114 (3)	0.0134 (4)	0.0117 (4)	0.0031 (2)	0.0061 (2)	0.0046 (2)
Mg	0.0107 (5)	0.0141 (6)	0.0101 (5)	0.0033 (4)	0.0042 (4)	0.0035 (4)
O1	0.0155 (8)	0.0170 (8)	0.0141 (8)	0.0036 (6)	0.0072 (6)	0.0041 (6)
O2	0.0152 (8)	0.0201 (8)	0.0171 (8)	0.0049 (6)	0.0096 (6)	0.0075 (6)
O3	0.0137 (8)	0.0277 (9)	0.0188 (8)	0.0081 (7)	0.0082 (6)	0.0131 (7)
O4	0.0128 (9)	0.0347 (10)	0.0299 (10)	0.0040 (7)	0.0037 (7)	0.0219 (9)
O5	0.0295 (9)	0.0169 (9)	0.0150 (8)	0.0016 (7)	0.0097 (7)	0.0052 (8)

### Geometric parameters (Å, º)

**Table:**

P—O1	1.5013 (16)	Mg—O5^ii^	2.0646 (18)
P—O2	1.5122 (15)	Mg—O5	2.0646 (18)
P—O3	1.5855 (16)	O3—H3	0.79 (4)
P—P^i^	2.1843 (12)	O4—H4A	0.82 (3)
Mg—O4^ii^	2.0580 (17)	O4—H4B	0.85 (4)
Mg—O4	2.0580 (17)	O5—H5A	0.75 (4)
Mg—O1	2.0637 (15)	O5—H5B	0.77 (4)
Mg—O1^ii^	2.0637 (15)		
			
O1—P—O2	116.02 (9)	O1^ii^—Mg—O5^ii^	88.52 (7)
O1—P—O3	112.90 (9)	O4^ii^—Mg—O5	90.25 (8)
O2—P—O3	106.05 (9)	O4—Mg—O5	89.75 (8)
O1—P—P^i^	108.73 (7)	O1—Mg—O5	88.52 (7)
O2—P—P^i^	108.36 (7)	O1^ii^—Mg—O5	91.48 (7)
O3—P—P^i^	104.04 (7)	O5^ii^—Mg—O5	180.0
O4^ii^—Mg—O4	180.0	P—O1—Mg	147.48 (9)
O4^ii^—Mg—O1	88.56 (7)	P—O3—H3	123 (2)
O4—Mg—O1	91.44 (7)	Mg—O4—H4A	128 (2)
O4^ii^—Mg—O1^ii^	91.44 (7)	Mg—O4—H4B	125.9 (19)
O4—Mg—O1^ii^	88.56 (7)	H4A—O4—H4B	106 (3)
O1—Mg—O1^ii^	180.00 (7)	Mg—O5—H5A	124 (3)
O4^ii^—Mg—O5^ii^	89.75 (8)	Mg—O5—H5B	121 (3)
O4—Mg—O5^ii^	90.25 (8)	H5A—O5—H5B	104 (4)
O1—Mg—O5^ii^	91.48 (7)		

Symmetry codes: (i) −*x*, −*y*+1, −*z*; (ii) −*x*, −*y*+2, −*z*+1.

### Hydrogen-bond geometry (Å, º)

**Table:**

*D*—H···*A*	*D*—H	H···*A*	*D*···*A*	*D*—H···*A*
O3—H3···O2^iii^	0.79 (4)	1.94 (4)	2.687 (2)	157 (3)
O4—H4*A*···O2^iv^	0.82 (3)	2.00 (3)	2.817 (2)	169 (3)
O4—H4*B*···O1^v^	0.85 (4)	1.94 (4)	2.786 (2)	173 (3)
O5—H5*A*···O2^vi^	0.75 (4)	2.03 (4)	2.768 (3)	165 (3)
O5—H5*B*···O3^vii^	0.77 (4)	2.08 (4)	2.829 (3)	165 (4)

Symmetry codes: (iii) *x*−1, *y*, *z*; (iv) *x*, *y*, *z*+1; (v) −*x*+1, −*y*+2, −*z*+1; (vi) *x*, *y*+1, *z*+1; (vii) −*x*, −*y*+2, −*z*.

## References

[bb1] Bloss, K. H., Henzel, N. & Beck, H. P. (1967). *Z. Anal. Chem.* **226**, 25–28.

[bb2] Brandenburg, K. (2012). *DIAMOND*. Crystal Impact GbR, Bonn, Germany.

[bb3] Collin, R. L. & Willis, M. (1971). *Acta Cryst.* B**27**, 291–302.

[bb4] Gjikaj, M., Wu, P. & Brockner, W. (2012). *Z. Anorg. Allg. Chem.* **638**, 2144–2149.

[bb5] Gjikaj, M., Wu, P. & Brockner, W. (2014). *Z. Anorg. Allg. Chem.* **640**, 379–384.

[bb6] Haag, J. M., LeBret, G. C., Cleary, D. A. & Twamley, B. (2005). *J. Solid State Chem.* **178**, 1308–1311.

[bb7] Hagen, S. & Jansen, M. (1995). *Z. Anorg. Allg. Chem.* **621**, 149–152.

[bb8] Leininger, E. & Chulski, T. (1953). *Inorg. Synth.* **4**, 68–71.

[bb9] Palmer, W. G. (1961). *J. Chem. Soc.* pp. 1079–1082.

[bb10] Ruflin, C., Fischbach, U., Grützmacher, H. & Levalois-Grützmacher, J. (2007). *Heteroat. Chem.* **18**, 721–731.

[bb11] Salzer, Th. (1878). *Liebigs Ann.* **194**, 28–39.

[bb12] Sheldrick, G. M. (2008). *Acta Cryst.* A**64**, 112–122. 10.1107/S0108767307043930 18156677

[bb13] Spek, A. L. (2009). *Acta Cryst.* D**65**, 148–155. 10.1107/S090744490804362X PMC2631630 19171970

[bb14] Stoe & Cie (1999). *X-SHAPE*. Stoe & Cie GmbH, Darmstadt, Germany.

[bb15] Stoe & Cie (2001). *X-RED*. Stoe & Cie GmbH, Darmstadt, Germany.

[bb16] Stoe & Cie (2002). *X-AREA*. Stoe & Cie GmbH, Darmstadt, Germany.

[bb17] Szafranowska, B., Ślepokura, K. & Lis, T. (2012). *Acta Cryst.* C**68**, i71–i82. 10.1107/S010827011204317X 23221237

[bb18] Szklarz, P., Chański, M., Ślepokura, K. & Lis, T. (2011). *Chem. Mater.* **23**, 1082–1084.

[bb19] Westrip, S. P. (2010). *J. Appl. Cryst.* **43**, 920–925.

[bb20] Wu, P., Wiegand, Th., Eckert, H. & Gjikaj, M. (2012). *J. Solid State Chem.* **194**, 212–218.

